# Fetal anogenital distance using ultrasound

**DOI:** 10.1002/pd.5459

**Published:** 2019-05-10

**Authors:** Ezra Aydin, Rosemary Holt, Daren Chaplin, Rebecca Hawkes, Carrie Allison, Gerald Hackett, Topun Austin, Alex Tsompanidis, Lidia Gabis, Shimrit Ilana Ziv, Simon Baron‐Cohen

**Affiliations:** ^1^ Autism Research Centre, Department of Psychiatry University of Cambridge Cambridge UK; ^2^ The Rosie Hospital Cambridge University Hospitals Foundation Trust Cambridge UK; ^3^ Child Development Centre Sheba Hospital Ramat Gan Israel; ^4^ CLASS Clinic Cambridgeshire and Peterborough Mental Health NHS Foundation Trust Cambridge UK

## Abstract

**Objective:**

This study measured anogenital distance (AGD) during late second/early third trimester of pregnancy to confirm previous findings that AGD can be measured noninvasively in the fetus using ultrasound and further showed differences in reference ranges between populations.

**Method:**

Two hundred ten singleton pregnancies were recruited at the Rosie Hospital, Cambridge, UK. A 2D ultrasound was performed between 26 and 30 weeks of pregnancy. AGD was measured from the centre of the anus to the base of the scrotum in males and to the posterior convergence of the fourchette in females.

**Results:**

A significant difference in AGD between males and females (*P* < .0001) was found, replicating previous results with a significant correlation between estimated fetal weight (EFW) and AGD in males only (*P* = .006). A comparison of AGD using reference data from an Israeli sample (n = 118) and our UK sample (n = 208) showed a significant difference (*P* < .0001) in both males and females, after controlling for gestational age (GA).

**Conclusion:**

Our results confirm that AGD measurement in utero using ultrasound is feasible. In addition, there are strong sex differences, consistent with previous suggestions that AGD is influenced by prenatal androgen exposure. AGD lengths differ between the UK and Israel; therefore, population‐specific normative values may be required for accurate clinical assessments.

## Abstract 2

What is already known about this topic?

- AGD is a sexually dimorphic measure.
- Previous research has linked AGD to prenatal androgen excess.
- This measure could assist with early identification of a range of neurodevelopmental, endocrine outcomes and the early diagnosis of conditions characterised by steroidogenic excesses or deficits.

What does this study add?

- This study confirms that, using ultrasound, AGD can be successfully measured in utero.
- We identify the need for population‐specific normative charts if this measure is to be used clinically and of diagnostic value.

**What is already known about this topic?**

- AGD is a sexually dimorphic measure.
- Previous research has linked AGD to prenatal androgen excess.
- This measure could assist with early identification of a range of neurodevelopmental, endocrine outcomes and the early diagnosis of conditions characterised by steroidogenic excesses or deficits.

**What does this study add?**

- This study confirms that, using ultrasound, AGD can be successfully measured in utero.
- We identify the need for population‐specific normative charts if this measure is to be used clinically and of diagnostic value.

**Abbreviations**
AGD: anogenital distance
EFW: estimated fetal weight

## 1 INTRODUCTION

Anogenital distance (AGD) refers to the length of the perineal area between the anus and genitals.^1^, ^2^, ^3^ AGD has been implicated as a predictor of androgen‐related outcomes in later life^4^ including reproductive^5^, ^6^ outcomes. There is growing interest in whether it may also predict neurodevelopmental outcomes associated with elevated prenatal steroids, such as autism.^7^, ^8^

In rodents, AGD length has been experimentally shown to be influenced by prenatal androgen exposure^3^ during the prenatal “masculinization programming window” (MPW).^9^, ^10^ AGD is highly sexually dimorphic in both animal^9^ and human^1^, ^2^, ^11^, ^12^ studies. The sexual dimorphism can be observed from as early as 11 to 13 weeks of fetal gestation in humans.^3^, ^12^ AGD continues to increase after birth and is correlated with birth weight,^11^, ^13^ and the differences observed in AGD prenatally are maintained across an individual's lifespan.^2^ Unlike with other proxy measures of early hormone exposure^14^ such as 2D:4D or penile length,^15^ postnatal hormonal exposure has not been found to further influence AGD^13^ in humans. This supports the theory that prenatal androgen exposure acts as the driver of AGD length. In adults, AGD correlates with circulating serum testosterone,^16^ as well as with the aromatisation ratio (of circulating testosterone to oestradiol).^17^ This suggests that AGD reflects the different aspects of the masculinisation pathways in development, and the relative balance between testosterone and oestrogens, rather than testosterone levels alone. It was thus suggested that AGD may be a suitable proxy to estimate prenatal androgen exposure.

Measuring AGD in 2D ultrasound scans has been shown to be feasible and reliable.^1^, ^12^, ^18^ This suggests that this measure has the potential to aid in the early identifications or understanding of pathogenesis of genitalia development,^12^ PCOS,^19^ and anorectal^4^, ^20^ and male genitalia^18^ malformation. However, there may be population‐specific differences in this measurement, with ethnicity or other regional factors affecting AGD centile charts and reference ranges.^1^ This study aims to further establish the feasibility of measuring AGD during the late second/early third trimester of pregnancy, for the first time in a UK sample, in particular to test for sex differences in AGD, and to assess population‐specific reference ranges, by comparing UK measures with those collected in Israel.

## 2 METHODS

### 2.1 Participants

Two hundred nineteen healthy fetuses, 104 male and 115 female, were recruited prospectively in the Rosie Maternity Hospital in Cambridge, UK. The same inclusion and exclusion criteria were used to those used in the Chaim Sheba Medical Centre study.^1^ Eligibility inclusion criteria for the study were as follows: pregnant women who were willing to have an additional ultrasound scan between 26 and 30 weeks of gestation (average GA: 28 weeks, SD = 1.25), with (a) little/no consumption of alcohol during pregnancy, (b) no smoking or recreational drug use during pregnancy, (c) a singleton appropriate‐for‐gestational age fetus, (d) the absence of any major fetal anomalies, and (e) fetus is not considered to have intrauterine growth restriction (IUGR) or be large for gestational age (LGA). Eligibility criterion for inclusion of the data in the final analysis was the birth of a clinically healthy baby. To observe difference in reference ranges, normal modelled AGD charts created from an Israeli population^1^ was used. The Israeli sample consisted of 424 healthy fetuses (218 female and 206 male fetuses) between 20 and 35 weeks of gestation. One hundred eighteen fetuses (59 male and 59 female) were between 26 and 30 weeks of gestation and used for this comparison.

### 2.2 Ethics

Ethical permission for the study was granted by the NHS, East of England Cambridge Central Research Ethics Committee (REC Ref 16/EE/0004), and the research and development department of Cambridge University Hospitals. All mothers gave written informed consent.

### 2.3 Procedure

**Figure 1:**
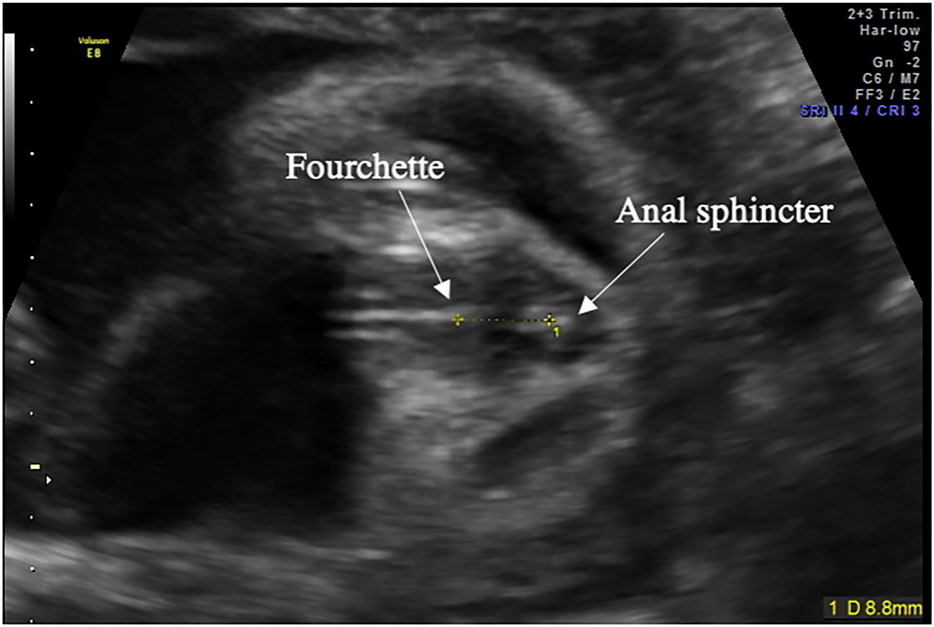
Example of the perineum in a female fetus demonstrating the anogenital distance measurement. AGD was measured from the centre of the anus to the posterior convergence of the fourchette. The posterior convergence of the fourchette was identified by the visibility of three white lines [Colour figure can be viewed at wileyonlinelibrary.com]

**Figure 2:**
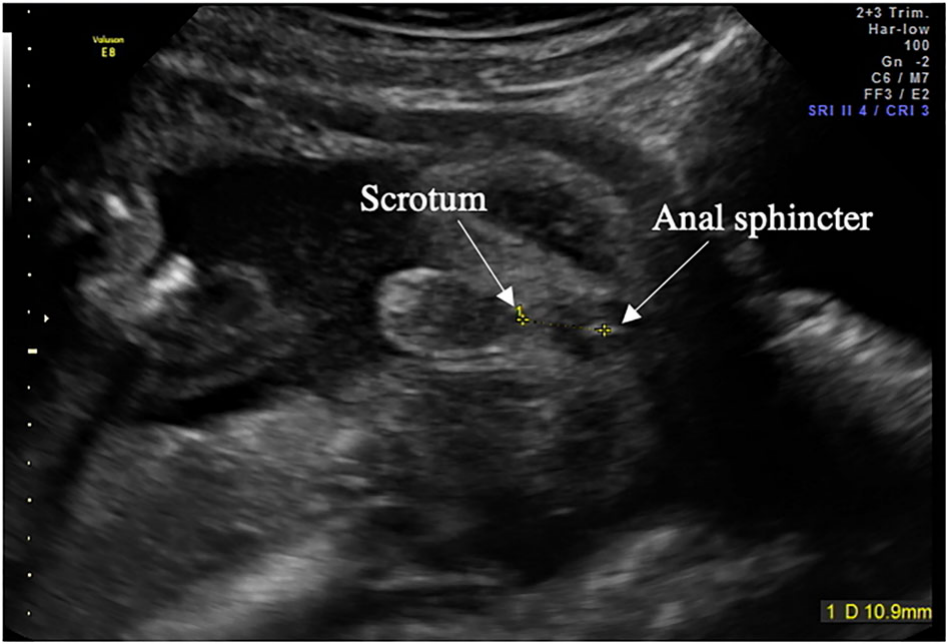
Example of the perineum in a male fetus demonstrating the anogenital distance measurement AGD was measured from the centre of the anus to the base of the scrotum. The scrotum was identified by the visibility of the scrotal sack [Colour figure can be viewed at wileyonlinelibrary.com]

Ultrasound scans were performed using a GE 8 Expert Ultrasound system (with a 4‐ to 8‐MHz curvilinear abdominal transducer). All women had completed a normal 20‐week anomaly scan and were made aware that this additional scan was for research purposes and was not a routine medical scan. Scans lasted approximately 40 minutes. During the scan, general fetal biometric measurements were also taken (ie, head circumference [HC], abdominal circumference [AC], and femur length [FL]) including dopplers to observe current fetal growth and check that the fetus had not become IUGR and LGA or developed any abnormalities since the last routine scan. AGD measurements were taken on the tangential section of the fetal perineum, where the anal sphincter would first be observed. AGD was measured from the centre of the anus to the base of the scrotum in males and to the posterior convergence of the fourchette in females using electronic callipers, following the same procedure used in previous research measuring fetal AGD^1^, ^12^ (Figures 1 and 2). For this measurement to be taken, the fetus' legs must be apart to accurately visualise the scrotum (in males) and fourchette (in females). If the legs were not separated when this measure was attempted, the mother was given cold water or asked to walk around for several minutes. In instances where the fetus was breech in presentation, the examination bed was tilted to move the fetus out of the pelvis and remove shadowing whilst the measurement was being taken. Failure to obtain any AGD measurement was only seen in 4.1% (nine fetuses, three male and six female; fetal positioning, four breech, four cephalic, and one not available) of the total sample (see Table 1 for population details). Because of the low failure rate of obtaining this measure, more invasive techniques such as transvaginal ultrasound and external cephalic version were not considered.

**Table 1:** Characteristics of the mothers and fetuses in the study

	Female (n = 115)	Male (n = 104)	All (n = 219)
	N (%)^c^	N (%)	N (%)
Ethnicity			
White	94 (81.7)	82 (78.8)	176 (80.4)
Black	1 (0.9)	2 (1.9)	3 (1.4)
East Asian	2 (1.7)	4 (3.8)	6 (2.7)
South Asian	2 (1.7)	3 (2.9)	5 (2.3)
Not Disclosed	16 (13.9)	13 (12.5)	29 (13.2)
Fetal position			
Breech	30 (26.1)	31 (29.8)	61 (27.8)
Cephalic	79 (68.7)	64 (61.5)	143 (65.3)
Transverse	2 (1.7)	2 (1.9)	4 (1.8)
Variable	2 (1.7)	2 (1.9)	3 (1.4)
Not Available	2 (1.7)	4 (3.8)	6 (2.7)

	Mean (SD)	Mean (SD)	Mean (SD)
Maternal Age	32.2 (4.3)	32.7 (4.8)	32.4 (4.5)
GA at time of scan	27.9 (1.25)	28.0 (1.25)	28.0 (1.25)
EFW^a^	1196.1 (228.3)	1238.6 (232.36)	1216.5 (230.7)

Birth information^b^	Female (n = 99)	Male (n = 92)	All (n = 191)
	N (%)	N (%)	N (%)
Delivery type			
SVD	63 (63.6)	46 (50)	109 (57)
C‐section	27 (27.3)	30 (32.6)	57 (30)
Assisted vaginal	9 (9.1)	15 (16.3)	24 (12. 5)
Vaginal breech	0 (0)	1 (1.1)	1 (0.5)

	Mean (SD)	Mean (SD)	Mean (SD)
Birth weight, g	3382.0 (540.4)	3460.9 (481.7)	3420.0 (513.16)
Gestation at birth, wk	39.6 (1.6)	39.6 (1.5)	39.6 (1.56)

a EFW data were not obtainable for four fetuses (one male, three female) because of fetal positioning.

b Birth data were not obtainable for 25 infants, as they gave birth at home or in another country.

c Percentages may not add up to 100 because of rounding.

Sex assignment was performed by observation of sonographic landmarks such as labial lines or fetal scrotum, in line with the established gold standard,^12^, ^21^ and was further confirmed at birth. As there was slight variability in AGD measurement due to placement of the fetus, several measurements (range 1‐3 freeze frame measurements) were taken and averaged for interobserver reliability purposes.

## 3 STATISTICS

### 3.1 Analysis

**Figure 3:**
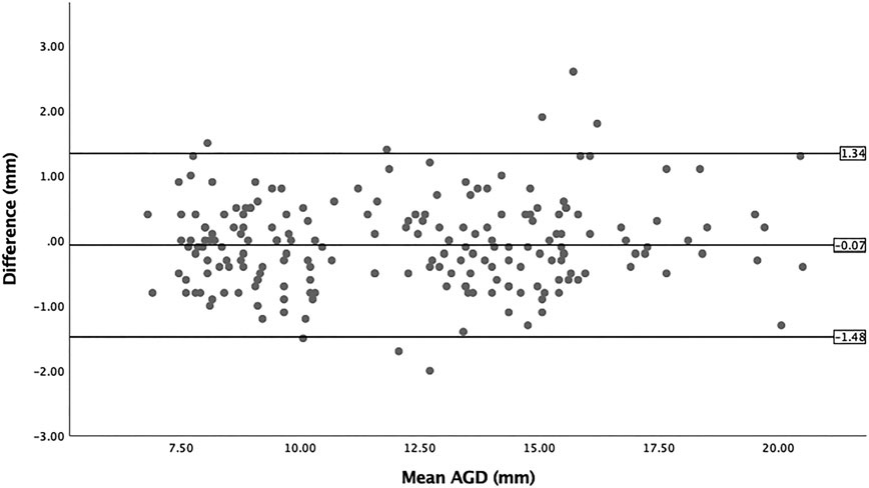
Bland‐Altman plot observing intraobserver variability

**Figure 4:**
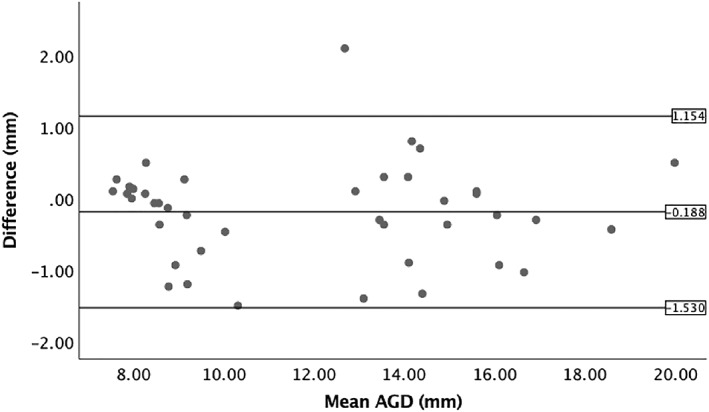
Bland‐Altman plot observing interobserver variability

SPSS statistical version 25 was used. Data were examined using a *t* test and a paired‐samples *t* test. To test for population differences (see Table 2 for raw AGD measurements of both populations), statistical analysis was performed using bilinear interpolation of the observed data from the UK set against an average fetal AGD biometry chart using the normal modelled AGD charts created from an Israeli population.^1^ This used the gestation and centiles to provide average expected AGD from a different population sample that were then gestation and centile matched to this UK population sample and analysed using a paired‐samples *t* test. As several freeze frame measurements were taken for AGD, intraobserver variability was assessed. The difference between the raw measures is presented in a Bland‐Altman plot (Figure 3). Linear regression showed no proportional bias (*P* = 0.753). Interobserver variability was assessed by comparing the mean measurements of two researchers on 20% of the sample. Each researcher was blind to the other's AGD measurements. The difference between the two mean measures is presented in a Bland‐Altman plot (Figure 4). Linear regression showed no proportional bias (*P* = .911). It was not possible to observe interobserver variability between Israel and the UK because of confidentiality and feasibility constraints. The effect of fetal position on the feasibility of AGD measurement was assessed with Pearson chi‐squared test.

**Table 2:** An overview of raw AGD (mm) measurements in Israel and UK cohorts

Week of Gestation	AGD Male Fetuses Mean ± SD (n)	AGD Female Fetuses Mean ± SD (n)	AGD Male Fetuses Mean ± SD (n)	AGD Female Fetuses Mean ± SD (n)
	Israel		United Kingdom	
26	14.1 ± 2.5 (16)	9.3 ± 2.0 (14)	13.4 ± 1.8 (19)	9.0 ± 2.0 (23)
27	14.5 ± 1.9 (12)	9.8 ± 1.6 (13)	14.5 ± 2.1 (26)	9.4 ± 1.8 (25)
28	16.3 ± 2.3 (10)	11.6 ± 2.0 (10)	16.2 ± 2.3 (26)	10.4 ± 1.9 (28)
29	18.5 ± 1.5 (11)	11.0 ± 1.3 (12)	15.1 ± 2.2 (21)	10.0 ± 2.5 (26)
30	17.9 ± 2.0 (10)	12.2 ± 2.3 (10)	14.9 ± 0.7 (9)	9.1 ± 2.0 (5)

*Note*. Data have been split by gestational week and separated by sex for both Israel and UK samples. Israel population data have been taken from Gilboa et al.^1^

Associations of AGD with maternal and fetal characteristics were assessed with univariate Pearson linear regression (Table 3) separately for each sex, to investigate potential covariates. The reported maternal body mass index (BMI) was calculated on weight and height measurements, as reported by the participants at the time of this additional scan. Nominally significant variables were then consecutively introduced to multivariate linear regression models, according to their level of significance, with AGD as the dependent variable and nominally significant characteristics as the independent variables (Table 4).

**Table 3:** Maternal and fetal characteristics and their effect on AGD in male and female fetuses, as assessed by Pearson linear regression

	Female		Male	
	Coefficient	P value	Coefficient	P value
Maternal characteristics				
Age	−0.09	0.38	0.13	0.32
BMI	−0.02	0.86	**0.25**	**0.03** *
Parity	−0.10	0.30	−0.05	0.60
Ethnicity	0.13	0.20	−0.01	0.93
PCOS	−0.03	0.73	0.02	0.83
IVF	0.11	0.27	−0.04	0.69
Fetal characteristics				
GA^a^	0.18	0.06	**0.25**	**0.01** *
EFW	0.15	0.13	**0.27**	**0.01** *
Birth weight	0.06	0.54	**0.25**	**0.02** *
Fetal position	0.19	0.054	0.17	0.09

*Note*. Asterisk denotes nominal association.

a GA: gestational age at the time of AGD measurement.

* Significant at the 0.05 level.

**Table 4:** Multivariate linear regression models, with AGD as the dependent variable

	Coefficient	Standard Error	P value
Model 1			
Sex	5.19	0.30	<.0001*
	***R*** ^**2**^ **: .60**		<.0001
Model 2			
Sex	5.05	0.30	<.0001*
EFW	0.00	0.00	.00*
	***R*** ^**2**^ **: .60**		<.0001
Model 3			
Sex	5.20	0.31	<.0001*
EFW	0.00	0.00	.00*
Birth weight	0.00	0.00	.14
	***R*** ^**2**^ **: .62**		**<.0001**
Model 4			
Sex	5.05	0.35	<.0001*
EFW	0.00	0.00	.00*
Birth Weight	0.00	0.00	.05*
Maternal BMI	0.06	0.039	.14
	***R*** ^**2**^ **: .64**		**<.0001**
Model 4			
Sex	5.10	0.35	<.0001*
EFW	−0.00	0.00	.48
Birth Weight	0.00		.01*
Maternal BMI	0.06	0.04	.10
GA^a^	0.76	0.41	.09
	***R*** ^**2**^ **: .65**		**<.0001** *

a GA: gestational age at the time of AGD measurement.

* Significant at the 0.05 level.

Data have been split by gestational week and separated by sex for both Israel and UK samples. Israel population data have been taken from Gilboa et al.^1^

## 4 RESULTS

The study included 210 fetuses (101 male and 109 female). Nine fetuses (three male and six female) were excluded from the analysis as adequate AGD measures could not be obtained because of fetal orientation during the ultrasound. There was no significant association between any of the specific fetal positions (eg, breech; see Table 1 for frequencies) and whether an adequate AGD measure could be obtained (Pearson chi‐squared test: *χ* ^2^ = 6.278, *P* = .393).

**Figure 5:**
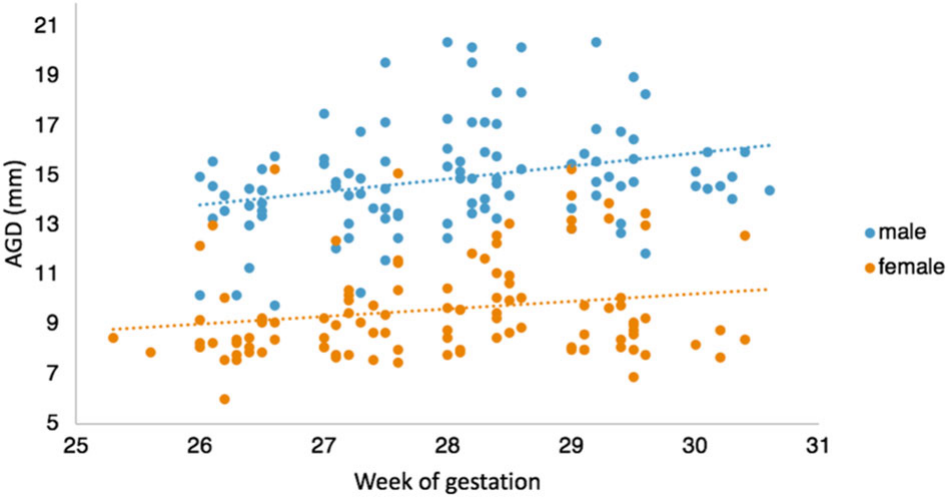
A scatterplot of raw AGD measures split by males fetuses and female fetuses between 25 and 31 weeks of gestation. Mean linear regression lines have been plotted by sex [Colour figure can be viewed at wileyonlinelibrary.com]

There was a significant difference in AGD between males (range: 9.80‐20.40 mm, mean: 14.90 mm) and females (range: 6.00‐15.30 mm, mean: 9.72 mm), *t* test: *t* = 17.406, [95% confidence interval (CI), 4.602‐5.775], df = 204.45, *P* < .0001. The raw AGD measurements split by sex is plotted in Figure 5.

Results from the univariate logistic regression analysis indicated that maternal BMI was significantly associated with AGD in male fetuses but not in females, whilst other maternal characteristics were not associate with AGD. The estimated fetal weight (EFW) of the fetus, GA, and the eventual birth weight were all associated separately to AGD in males but not in female fetuses.

Data from 210 fetuses were analysed (101 male and 109 female) to test for a correlation between EFW and AGD. There was a partial significant correlation between EFW and AGD (*r* = .193, *P* = .005); however, when the analysis was split by sex (females, *r* = .135, *P* = .162; males, *r* = .272, *P* = .006), this correlation only remained significant in males.

Variables that were associated with AGD (Table 3) were included in multivariate linear regression models. The maximum variance that could be explained by all predictors was 64.5% (adjusted *R* ^2^: .645, *P* < .0001), with fetal sex having the most pronounced effect on AGD, across all models (Table 4).

**Figure 6:**
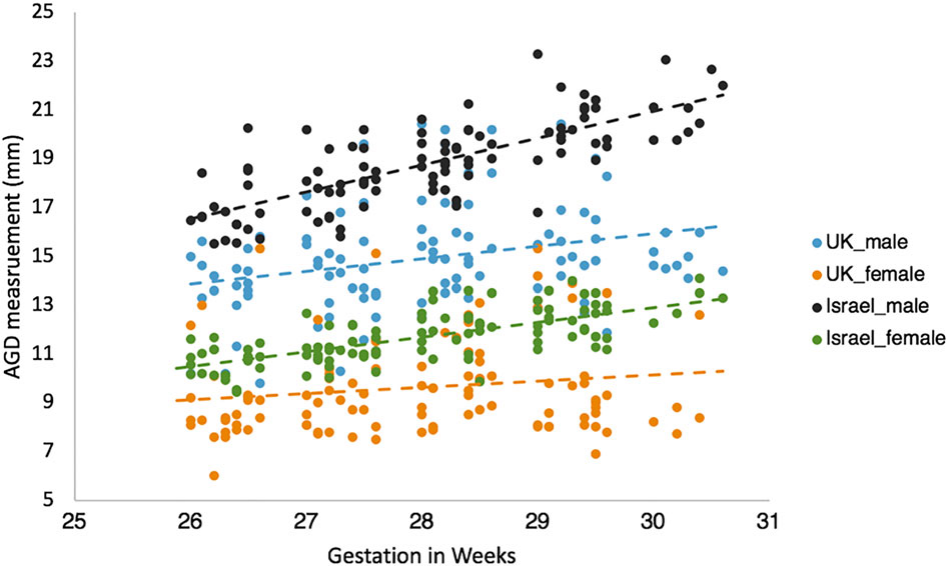
A scatterplot of matched raw AGD measures taken in both UK and Israel populations by sex between 26 and 31 weeks of gestation. Mean linear regression lines have been plotted by sex [Colour figure can be viewed at wileyonlinelibrary.com]

A comparison of AGD between 26 and 30 weeks of gestation in an Israeli sample and our UK sample showed a significant difference (*t* = 17.214, [95% CI, 2.606‐3.280], *P* = .000, *d* = 1.21) in both males and females, after controlling for GA (weeks and days): females (*t* = 9.489, [95% CI, 1.596‐2.393], *P* < .0001, *d* = .93) and males (*t* = 16.80, [95% CI, 3.461‐4.389] *P* < .0001, *d* = 1.70). This sample included 202 fetuses (98 male and 104 female). Two males and five females from the UK sample were not included in this analysis as centile measurements from their ultrasound were not obtained. See Table 5 for means and SD of the two populations (Figure 6).

**Table 5:** Mean and SD of raw AGD measurements (mm) from both Israel and UK samples

	All	Male	Female
	Mean (SD)		
Israel AGD	15.09 (3.86)	18.78 (1.75)	11.63 (1.06)
UK AGD	12.15 (3.35)	14.85 (2.18)	9.61 (1.98)

*Note*. All gestational weeks combined.

## 5 DISCUSSION

This study supports previous findings that AGD can be reliably measured in utero during the second and third trimesters of pregnancy. The minimal variability in this measurement between independent raters suggests that this can be measured reliably by both sonographers and researchers. In addition, we replicated previous fetal AGD results showing AGD is strongly sexually dimorphic as early as the late second trimester of pregancy.^1^ Finally, this study revealed a variation in AGD measurement ranges between the UK and Israel.

Previous research has demonstrated the feasibility of reliably measuring anogenital distance from 21 weeks of gestation.^1^ Research looking at newborn,^2^, ^11^ infant,^2^, ^22^ and adult^23^, ^24^ AGD has linked this measure to a range of reproductive and developmental outcomes in later life. As a result, researchers have suggested the potential use of AGD length in linking fetal programming to adult conditions. Early identification of predispositions to later diagnoses (eg, PCOS) or postnatal outcomes (ie, genital anomalies) could help inform both early pharmacological and psychological treatment. The ability to reliably measure AGD in utero shows this measure has potential to be introduced as part of a routine ultrasound scan.

Neonatal and animal research shows a slight correlation between weight and AGD measurement.^11^, ^25^ We found a similar significant correlation to EFW and birth weight (Table 3) in males but not in female fetuses. This could be due to sexual dimorphism in the regulation of growth,^26^ or it may be because the range of AGD is greater defined in males, making it easier to detect the correlation. In addition, we noted a modest association of maternal BMI at the time of the ultrasound scan (mean GA: 28 weeks) with AGD in males. This may be attributed to fetal weight as well, since the effect was not detected after combining maternal BMI with measures of fetal weight (Table 4).

Previous research has suggested potential differences in AGD means and ranges between ethnic backgrounds.^1^ The influence of biological and environmental factors on prenatal growth has been well documented.^27^, ^28^, ^29^ We found significant variation between our UK sample and gestational growth charts from a sample in Israel. This demonstrates that similar to fetal biometry charts (such as femur length and head circumference), there is population‐specific variability in AGD. The observed variation in AGD length between our UK sample and Israel samples could be related to differences in fetal positioning during the ultrasound. As the same anthropometric protocol was implemented (ie, initial identification of the anal sphincter) to guide the measurement and use of the same ultrasound plane, this should result in little to no variability in the measurement taken between the two sites. Alternatively, differences in genetic predisposition to congenital adrenal hyperplasia in Israel could account for the observed variation in measured distances.^30^ Causes of this variability would need to be investigated, but given its existence, population‐specific charts need to be created if this measure is to be used clinically or for research purposes.

The introduction of reliable and reproducible ultrasound measures such as AGD will help further our understanding of the role of the intrauterine environment, fetal reproductive programming, and its influences on later adult outcomes through noninvasive studies. Whilst direct clinically relevant outcomes remain to be thoroughly explored, the sexually dimorphic nature and feasibility of prenatal AGD measurement demonstrates the potential to assist in early diagnosis for a number of outcomes (ie, male genital malformation, PCOS, and other sexually dimorphic developmental conditions).

There are some limitations to this study, including the size of the sample and the narrow GA range of 26 to 30 weeks of gestation. It was not possible to coax the fetus into a more optimal position for AGD measurement for n = 9 (4.1%), using noninvasive techniques (ie, cold water). Because of time and resource constraints, mothers were not invited back for a second attempt at taking the measurement. Future studies may consider the use of more invasive techniques including transvaginal ultrasound or external cephalic version to obtain this measure in fetuses where noninvasive methods are unsuccessful. Research has suggested that maternal anxiety has an influence on fetal and infant maturation.^31^ At the time of scanning, no fetus showed IUGR, was LGA, or had a lower than expected EFW; therefore, maternal anxiety was not controlled for in this study. Additionally, socio‐economic status and education levels were not collected. Lastly, it was not possible to measure interobserver variability between the two countries (UK and Israel). Future studies are needed to observe population differences in this measure as well as longitudinal change from postnatal to adult life.

## 6 CONCLUSIONS

Overall, we show significant prenatal sexual dimorphism and demonstrate the ability to successfully measure AGD in utero. These results support the potential for this measure to be utilised in the prediction of later outcomes. We also suggest the need for large scale population‐specific normative charts if this measure is to be used to inform research or used clinically. In order to fully assess the utility of prenatal AGD as a clinical measure, longitudinal research from prenatal to adult life is needed. We will continue to follow this cohort longitudinally and assess and compare the feasibly and utility of measuring AGD length at prenatal and infant stages of development.

## CONFLICT OF INTEREST

The authors have no potential conflicts of interest to disclose.

## FUNDING INFORMATION

This research was funded by a grant from the National Institute of Health Research (NIHR) Senior Investigator Award to S.B.C. and grants to S.B.C. from the Medical Research Council (MRC), the Wellcome Trust, and the Autism Research Trust (ART). The research was conducted in association with the National Institute for Health Research (NIHR) Cambridge Biomedical Research Centre and the NIHR Collaboration for Leadership in Applied Health Research and Care East of England at Cambridgeshire and Peterborough NHS Foundation Trust. The views expressed are those of the authors and not necessarily those of the NHS, the NIHR, or the Department of Health and Social Care. This research was possible because of two applications to the UK Biobank: Projects 20904 and 23787. The project leading to this application has received funding from the Innovative Medicines Initiative 2 Joint Undertaking (JU) under grant agreement no. 777394. The JU receives support from the European Union's Horizon 2020 research and innovation programme and EFPIA and AUTISM SPEAKS, Autistica, SFARI. This work also received support from the Templeton World Charitable Foundation.

## FINANCIAL DISCLOSURE

All authors have indicated that they have no financial relationships relevant to this article to disclose.

## DATA AVAILABILITY STATEMENT

The data that support the findings of this study are available on request from the corresponding author. The data are not publicly available due to privacy or ethical restrictions.
